# Preparation and application of a *Brucella* multiepitope fusion protein based on bioinformatics and Tandem Mass Tag-based proteomics technology

**DOI:** 10.3389/fimmu.2024.1509534

**Published:** 2025-01-10

**Authors:** Qi Wu, Yuan Yuan, Liping Guo, Yujia Xie, Meixue Yao, Dehui Yin

**Affiliations:** ^1^ Jiangsu Engineering Research Center of Biological Data Mining and Healthcare Transformation, Xuzhou Medical University, Xuzhou, China; ^2^ Zhuhai People’s Hospital (The Affiliated Hospital of Beijing Institute of Technology, Zhuhai Clinical Medical College of Jinan University), Zhuhai, China; ^3^ Key Laboratory of Human Genetics and Environmental Medicine, Xuzhou Medical University, Xuzhou, China; ^4^ Xuzhou Engineering Research Innovation Center of Biological Data Mining and Healthcare Transformation, Xuzhou Medical University, Xuzhou, China; ^5^ Center for Medical Statistics and Data Analysis, Xuzhou Medical University, Xuzhou, China

**Keywords:** brucellosis, diagnosis, multiepitope fusion protein, bioinformatics, proteomics

## Abstract

**Introduction:**

Brucellosis is a widespread zoonotic disease that poses a considerable challenge to global public health. Existing diagnostic methods for this condition, such as serological assays and bacterial culture, encounter difficulties due to their limited specificity and high operational complexity. Therefore, there is an urgent need for the development of enhanced diagnostic approaches for brucellosis.

**Methods:**

Tandem mass tag (TMT) proteomic analysis was conducted on the wild-type strain *Brucella abortus* (*B. abortus*) DT21 and the vaccine strain *B. abortus* A19 to identify proteins with high expression levels. The proteins that exhibited high expression in the wild-type strain were selected based on the proteomic results. Subsequently, B-cell linear epitopes were predicted using multiple computational tools, including ABCpred, SVMTriP, BCPred, and Bepipred Linear Epitope Prediction 2.0. These epitopes were concatenated to construct a multiepitope fusion protein. Following prokaryotic expression and purification, an indirect enzyme-linked immunosorbent assay (iELISA) was developed. A total of 100 positive serum samples, 96 negative serum samples, and 40 serum samples from patients infected with other pathogens were collected and analyzed using the established iELISA. Furthermore, the protein was assessed for its capability to differentiate human brucellosis from lipopolysaccharide (LPS).

**Results:**

Proteomic analysis revealed the presence of 152 proteins with high expression levels in the wild-type strains. A multiepitope fusion protein, comprising a total of 32 predicted B-cell linear epitopes, was successfully prepared. The results from the iELISA indicated that the multiepitope fusion protein exhibited exceptional diagnostic performance, evidenced by an area under the receiver operating characteristic curve (AUC) of 0.9576, a sensitivity of 0.9300, and a specificity of 0.8542. In comparison to the commonly utilized LPS antigen, the fusion protein demonstrated a reduced level of cross-reactivity.

**Conclusions:**

A novel multiepitope fusion protein has been successfully developed utilizing bioinformatics and TMT proteomics technology. This fusion protein demonstrates significant potential as a diagnostic antigen for brucellosis, exhibiting high sensitivity and specificity.

## 1 Introduction

Brucellosis is a longstanding global zoonosis that has been reported in over 170 countries and regions, with an estimated 500,000 new cases occurring annually worldwide. This disease represents a significant public health challenge on a global scale, garnering substantial attention within the public health sector (1). Presently, in China, the incidence of brucellosis is on the rise, attributed to rapid economic development and the increasing movement of animals and animal products. In 2023, the number of new cases in the country surpassed 70,400 (2, 3).

Despite advancements in existing diagnostic methods, including serological, pathogenetic, and molecular biology testing, there remain significant limitations in the practical application of each of these approaches (4, 5). Serological methods, such as the standard agglutination test (SAT), the rose bengal plate agglutination test (RBPT), and the enzyme-linked immunosorbent assay (ELISA), are widely employed in clinical diagnostics due to their operational simplicity and cost-effectiveness. However, these methods are not without their drawbacks. For instance, the specificity of serological diagnoses can be adversely affected by antigenic factors. A frequently utilized antigen, lipopolysaccharide (LPS), is susceptible to cross-reactivity with *Yersinia enterocolitica* O9, potentially leading to false-positive results. Furthermore, while pathogenicity tests are known for their high accuracy, they are complex to execute and present challenges for implementation in primary care settings (6–9).

Bioinformatics, defined as the integration of biology, computer science, and information technology, plays a crucial role in contemporary biomedical research, particularly within the domains of genomics and proteomics (10). This interdisciplinary field encompasses the development and application of computational tools and algorithms designed to analyze and interpret complex biological data, including DNA sequences, protein structures, and gene expression profiles. In the realm of vaccine design, bioinformatics has become essential, facilitating the identification of potential antigens, the prediction of immune responses, and the optimization of vaccine candidates (11). Through the analysis of genomic sequences, bioinformatics aids researchers in understanding the pathogenicity and antigenic characteristics of microorganisms, which is vital for the development of effective vaccines. Furthermore, the analysis of proteomic data using bioinformatics tools can identify key proteins that are differentially expressed in pathogenic strains, thereby assisting in the selection of specific epitopes for vaccine development. The application of bioinformatics in these contexts has resulted in significant advancements in the understanding of host-pathogen interactions and the rational design of vaccines with enhanced efficacy and safety (12).

This study addresses the current status and challenges associated with the diagnosis of brucellosis by employing proteomics and bioinformatics to develop multiepitope fusion proteins. The research involves a meticulous screening of key antigenic epitopes derived from wild-virulent strains of *Brucella*, which are subsequently fused into a single protein molecule. The objective is to construct a novel diagnostic antigen characterized by high specificity, aimed at facilitating the development of an indirect ELISA (iELISA) for the diagnosis of human brucellosis.

## 2 Materials and methods

### 2.1 Serum samples and bacterial strains

A total of 100 positive and 96 negative serum samples were obtained from the Xuzhou Center for Disease Control and Prevention, all of which were confirmed as either positive or negative through the SAT. Furthermore, serum samples from 40 febrile patients infected with various pathogens (stored in the laboratory; detailed information is provided in **Supplementary Material 1**: Cross-Reactivity Assessment) were utilized to assess the cross-reactivity of the developed method. Additionally, the vaccine strain *Brucella abortus* A19 and the wild-type *Brucella abortus* DT21, both isolated and preserved by the China Animal Health and Epidemiology Center, were employed in this study.

### 2.2 TMT proteomics

#### 2.2.1 Bacterial culture

The preserved bacterial strain was inoculated into 500 mL of tryptic soy broth (TSB) medium and incubated at 37°C with shaking for a duration of 24 to 48 hours. After the incubation period, 5 mL of 1% formaldehyde was introduced to inactivate the bacteria, which were subsequently stored at 4°C for future use.

#### 2.2.2 TMT proteomics analysis

TMT proteomics analysis was conducted in accordance with established protocols documented in the literature. This process encompassed several key steps, including protein extraction and quantification, protein digestion and TMT labeling, as well as LC-MS/MS analyses, followed by both qualitative and quantitative assessments of the proteins (13).

### 2.3 Preparation of fusion proteins

#### 2.3.1 Prediction of B-cell linear epitopes

Based on the results obtained from TMT proteomics, proteins that exhibited high expression levels in the wild-type strain were selected for further analysis. The amino acid sequences of these proteins were retrieved from the NCBI protein database (http://www.ncbi.nlm.nih.gov/protein/). To enhance the accuracy of epitope prediction, four B-cell linear epitope prediction tools were employed: ABCpred (https://webs.iiitd.edu.in/raghava/abcpred/index.html, with a default threshold of 0.5), SVMTriP (http://sysbio.unl.edu/SVMTriP, with no threshold), BCPred (http://ailab-projects2.ist.psu.edu/bcpred/predict.html, with a default specificity threshold of 75%), and Bepipred Linear Epitope Prediction 2.0 (http://tools.iedb.org/bcell/, with a default threshold of 0.5) (14–17). The predicted B-cell epitopes from all tools were compared, and overlapping B-cell epitopes were selected as candidate epitopes. For each prediction tool, the prediction threshold was maintained at the default value, with the exception of SVMTriP, and epitopes with scores exceeding 0.5 were considered potential candidates.

#### 2.3.2 Construction of the fusion protein amino acid sequence

The predicted linear epitopes of B-cells were concatenated, incorporating a ‘GS’ linker between adjacent epitopes to construct the amino acid sequence of the fusion protein. This sequence was subsequently submitted to Beijing Protein Innovation Co., Ltd. for codon optimization, thereby rendering it suitable for prokaryotic expression. Gene synthesis was conducted, and a 6×His tag was incorporated to facilitate subsequent protein purification. The three-dimensional (3D) molecular model of the fusion protein was predicted using I-TASSER (https://zhanggroup.org/I-TASSER/), while the antigenicity of the fusion protein was assessed using VaxiJen (http://www.ddg-pharmfac.net/vaxijen/VaxiJen/VaxiJen.html), employing a default threshold of 0.4.

#### 2.3.3 Prokaryotic expression of the fusion protein

The gene encoding the synthesized fusion protein was subsequently cloned and inserted into the expression vector pET30a. This vector was then transformed into competent BL21 cells to facilitate IPTG-induced expression. The transformation process involved several steps. Initially, the competent BL21 cells were stored at -80°C and were slowly thawed on ice. They were then mixed with the pET30a vector and incubated on ice for 30 minutes. Following this, the samples underwent a heat shock at 42°C for 90 seconds, after which they were immediately cooled on ice for 2 minutes. Subsequently, 800 μL of LB medium was added to the mixture, which was incubated at 37°C for 45 minutes before being centrifuged at 5,000 rpm for 3 minutes. Most of the supernatant was discarded, leaving approximately 100–150 μL, after which the cells were resuspended. The resulting suspension was plated onto LB agar plates containing the appropriate antibiotic and incubated overnight at 37°C. The cultured bacterial mixture was then transferred to 250 mL of LB liquid medium supplemented with the corresponding antibiotic and incubated at 37°C with shaking at 200 rpm until the optical density at 600 nm (OD600) reached 0.6–0.8. Expression of the target protein was induced by the addition of 0.5 mM IPTG, followed by incubation at 37°C for 4 hours. The mixture was subsequently centrifuged at 8,000 rpm for 6 minutes, and the supernatant was discarded to collect the cells. The resulting pellet was resuspended in 20–30 mL of 10 mM Tris-HCl (pH 8.0) solution, and ultrasonic disruption was performed (500 W, 180 cycles, 5 seconds per cycle, with 5-second intervals). One hundred microliters of the disrupted bacterial suspension was then centrifuged at 12,000 rpm for 10 minutes. Following centrifugation, 50 μL of the supernatant was transferred to a separate Eppendorf tube, and the pellet was resuspended in 50 μL of 10 mM Tris-HCl (pH 8.0) solution. A 12% SDS-PAGE gel was employed to ascertain the presence of the target protein in either the supernatant or the pellet for subsequent purification.

#### 2.3.4 Fusion protein purification

The nickel affinity chromatography column (Ni Sepharose 6 Fast Flow, GE Healthcare) was initially washed with deionized water until the pH stabilized at 7.0. Subsequently, the column was equilibrated with approximately 100 mL of a 10 mM Tris-HCl buffer (pH 8.0). This was followed by further equilibration using approximately 50 mL of the same buffer supplemented with 0.5 M NaCl. The sample containing the target protein was then diluted and applied to the column. Following the loading of the sample, the column was washed with a 10 mM Tris-HCl buffer (pH 8.0) containing 0.5 M NaCl. The target protein was eluted using 10 mM Tris-HCl buffers (pH 8.0) containing imidazole concentrations of 15 mM, 60 mM, and 300 mM, along with 0.5 M NaCl. The eluted protein peaks were collected, and the purification efficiency was assessed through 12% SDS-PAGE electrophoresis. Additionally, the protein content was quantified using a BCA protein quantification kit.

### 2.4 Establishment of iELISA and serum detection

The iELISA was conducted according to the following protocol. Initially, the purified protein was diluted in carbonate-bicarbonate buffer solution (CBS) to achieve a concentration of 10 µg/mL, and 100 µL of this solution was dispensed into each well of a 96-well microplate (Corning, USA). The microplate was then incubated overnight at 4°C. Following incubation, the wells were washed three times with PBST. Subsequently, 300 µL of blocking solution (5% skim milk in PBS) was added to each well, and the samples were incubated at 37°C for 2 hours. After another round of washing with PBST, human serum diluted in PBS (1:200) was introduced and incubated at 37°C for 1 hour. Following three additional washes with PBST, 100 µL of horseradish peroxidase (HRP)-conjugated rabbit anti-human IgG (diluted 1:10,000, Thermo Fisher, USA) was added to each well and incubated at 37°C for 1 hour. The plate was washed three times with PBST, after which the tetramethylbenzidine (TMB) substrate solution was added, and the plate was incubated in the dark for 10 minutes to allow for color development. The reaction was terminated by the addition of 2 M H_2_SO_4_, and the optical density at 450 nm (OD450) was measured using a microplate reader (Versa Max microplate reader, MD, USA). Laboratory-stored LPS (3 mg/mL, provided by the China Animal Health and Epidemiology Center) served as a control antigen, and serum samples were analyzed in triplicate following the same procedure. The sensitivity, specificity, area under the curve (AUC), and cutoff values were determined through receiver operating characteristic (ROC) curve analysis.

### 2.5 Cross-reactivity assessment

Following the aforementioned iELISA procedure, sera from febrile patients without brucellosis were analyzed by using the two antigens to evaluate the cross-reactivity of the constructed fusion protein. Cross-reactivity was assessed based on the cut-off value determined by the ROC curve.

### 2.6 Statistical methods

Dot plot and ROC curve analyses were conducted using GraphPad Prism version 6.05. Statistical analyses were performed utilizing one-way analysis of variance (ANOVA) and Student’s t-test (unpaired t-test), with a significance level established at p < 0.05.

## 3 Results

### 3.1 Proteomics analysis

Through TMT quantitative analysis, a total of 152 proteins exhibiting high expression levels were identified in the wild-type strain, while 102 highly expressed proteins were identified in the vaccine strain (see **Supplementary Material 2**). From the highly expressed proteins in the wild-type strain, seven target proteins were selected for the prediction of B-cell epitopes (refer to **Table 1**).

**Table 1:** Protein information selected based on TMT proteomics results.

Accession	Protein	Score Sequest HT	#Unique Peptides	#Peptides	MW [kDa]	-10lg
Q57AX1	Malate dehydrogenase	86.08	3	3	33.7	−∞
P34939	Chaperonin GroEL	157.22	1	5	57.8	73.06
A5G1G2	Chaperonin GroEL	25.6	1	3	58.2	57.85
P66827	Superoxide dismutase [Cu-Zn]	188.52	4	4	18.3	50.07
A5VTU2	Cochaperonin GroES	23.46	3	3	10.3	148.12
Q05981	Chaperone protein DnaK	375.3	4	9	68.2	45.63
B8DT62	Chaperone protein DnaK	4.89	1	1	66.7	38.15
Q6G554	Chaperone protein DnaK	149.27	1	5	68.2	36.89
Q28VY3	Chaperone protein DnaK OS=Jannaschia sp. (strain CCS1)	14.87	1	3	68.9	33.63
Q8FXF9	HTH-type quorum sensing-dependent transcriptional regulator VjbR	83.93	5	5	28.6	60.90
Q2YK66	Putative ABC transporter peptide-binding protein BAB2_0812	28.47	1	1	57.3	33.19

### 3.2 Epitope prediction

Based on the results obtained from proteomics and a comprehensive review of the pertinent literature, specific proteins were identified as potential candidate targets. A total of 32 epitopes were predicted, as presented in **Table 2**. Subsequently, these epitopes were concatenated to create the amino acid sequence of the fusion protein using a linker.

**Table 2:** Predicted candidate epitope information.

Protein (sub-cellular localization*)	Accession	Epitope (amino acid sequence)	Start-end position	Length
Malate dehydrogenase (Unknown)	Q57AX1	GTPQGKGLDIAESSPVDGFDA	38-58	21
		GVPRKPGMS	80-88	9
		AGIKKYAP	105-112	8
		GWTSQDKLD	201-209	9
		ERIIEIDLDKDEKAQF	283-298	16
Chaperonin GroEL (Cytoplasmic)	P34939	VREVASKTNDI	74-84	11
		RAKKVSISK	319-327	9
		IEETTSDYDREK	353-364	12
		VKGANDDQEA	428-436	9
		DKNEDNFGYNAQTSEY	470-485	16
		KDAPAGMPGGM	527-537	11
Chaperonin GroEL (Cytoplasmic)	A5G1G2	ETEVKERKDR	386-395	10
		KKAPAGGDA	528-536	9
Superoxide dismutase [Cu-Zn] (Periplasmic)	P66827	NPSCAPGEKDGKIV	72-84	13
		NTHHHLGPEGDG	98-109	12
Co-chaperonin GroES (Cytoplasmic)	A5VTU2	RVIVRRVESE	12-21	10
		AGDRVLFGKWSGTE	64-77	14
Chaperone protein DnaK (Cytoplasmic)	Q05981	MVTKDKDLVPYKIVKG	81-96	16
		AFFGKEPHKGV	350-360	11
		EAAQAAEGAGA	599-609	11
Chaperone protein DnaK (Cytoplasmic)	B8DT62	DKGTGKE	469-475	7
		KEEIDQMIK	488-496	9
Chaperone protein DnaK (Cytoplasmic)	Q6G554	QSFFGKDPHKGVNP	349-358	10
		QGEREMANDNKLL	437-449	13
		MVKDAEEHAAEDKK	510-523	14
		YEASQAATPNTE	598-609	12
Chaperone protein DnaK (Cytoplasmic)	Q28VY3	TKFFGKEPHKG	349-359	11
		SLEEHGEKVDP	544-554	11
HTH-type quorum sensing-dependent transcriptional regulator VjbR (Unknown)	Q8FXF9	WVARYSSKN	69-77	9
		IHGTVCGCKDANS	173-185	13
Putative ABC transporter peptide-binding protein BAB2_0812 (Periplasmic)	Q2YK66	SNDVSTFS	254-261	8
		IWTPAPAGGP	506-515	10

*Sub-cellular localization predicted by PSORTb (https://www.psort.org/psortb/).

### 3.3 Fusion protein preparation

**Figure 1:**
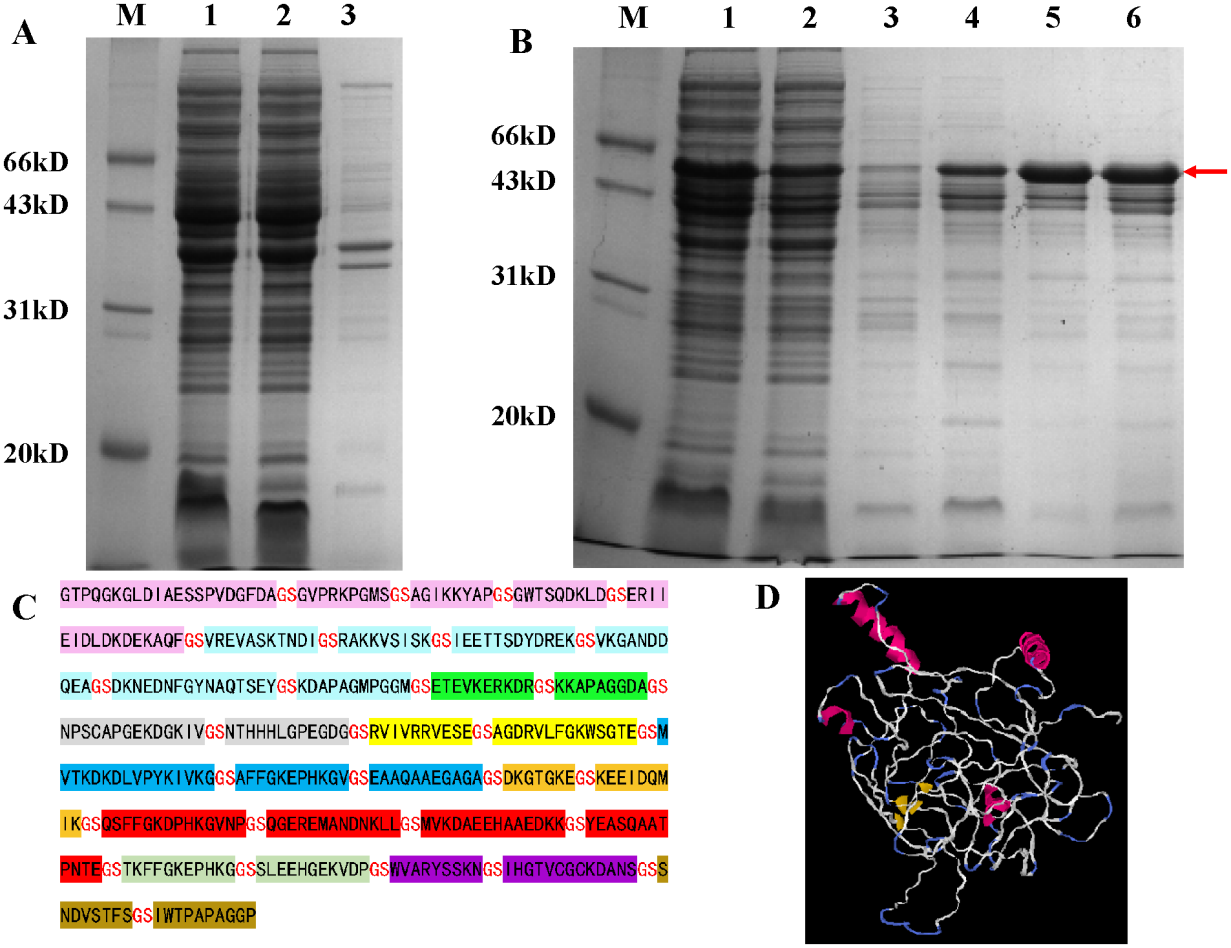
12% SDS-PAGE analysis of fusion protein prokaryotic expression results. **(A)** Detection of fusion protein expression. M: marker; Lane 1: whole-cell lysate after sonication; Lane 2: supernatant after sonication; Lane 3: pellet after sonication. **(B)** Results of large-scale expression and purification of the fusion protein. M: marker; Lane 1: crude protein sample; Lane 2: flow-through fraction; Lane 3: elution with 15 mM imidazole; Lane 4: elution with 60 mM imidazole; Lanes 5 and 6: elution with 300 mM imidazole; **(C)** Amino acid sequence of the fusion protein; **(D)** 3D structural models of fusion proteins predicted by I-TASSER.

Following prokaryotic expression, the target protein was identified in the supernatant. Subsequent purification of the fusion protein resulted in a purity level of 90.1%. The findings are illustrated in **Figure 1**. The prediction from VaxiJen indicated an antigenicity score of 1.052, suggesting that it is a probable antigen.

### 3.4 Results of iELISA

**Figure 2:**
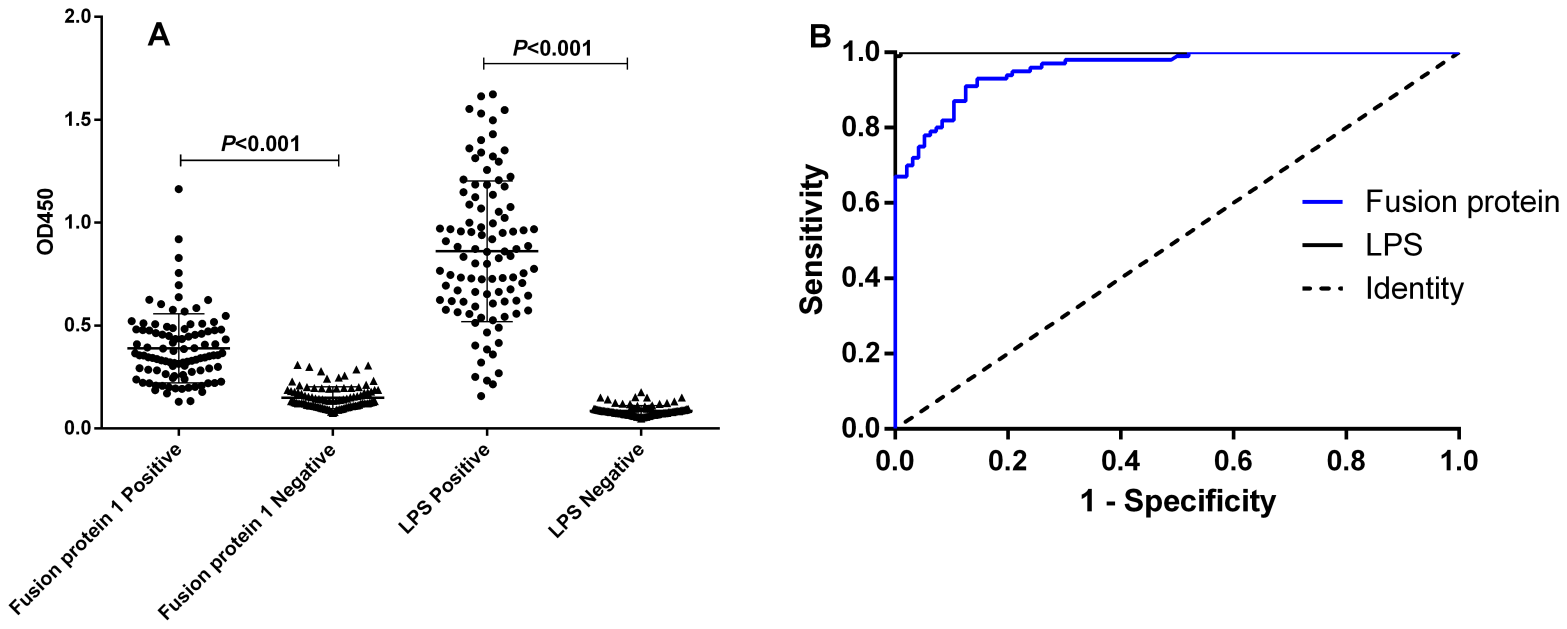
I-ELISA analysis of human serum samples. **(A)** Dot plot of human serum samples. **(B)** ROC analysis of human sera.

The analysis of the ROC curve revealed that the area under the diagnostic curve (AUC) for the fusion protein was 0.9576 (95% CI, 0.9337–0.9814), while the AUC for LPS was 0.9999 (95% CI, 0.9995–1.000). These findings indicated that both antigens possess excellent diagnostic value. Utilizing the Youden index, the cutoff value for the diagnosis using the fusion protein was established at 0.1970. At this threshold, the sensitivity and specificity of the diagnostic method were found to be 0.9300 (95% CI, 0.8611–0.9714) and 0.8542 (95% CI, 0.7674–0.9179), respectively. Conversely, the cutoff value for the diagnosis using LPS was determined to be 0.1953, with a sensitivity of 0.9900 (95% CI, 0.9455–0.9997) and a specificity of 1.000 (95% CI, 0.9623–1.000). The detailed results are illustrated in **Table 3**, **Figure 2**, and the **Supplementary Material**.

**Table 3:** Positive and negative predictive values of the test calculated for different cutoff values.

Antigen	Cutoff value	Positive		Negative		Accuracy (%)	PPV (%)	NPV (%)
		TP	FN	TN	FP			
Fusion protein	>0.1970	93	7	82	14	89.29	86.92	92.13
LPS	>0.1953	99	1	96	0	99.49	100.0	98.97

TP, true positive; TN, true negative; FP, false-positive; FN, false-negative; accuracy, (TP+TN/TP+FN+TN+FP) ×100; PPV, positive predictive value (TP/TP+FP) ×100; NPV, negative predictive value (TN/TN+FN) ×100.

### 3.5 Cross-reactivity assessment

Utilizing the iELISA and the established cutoff values, cross-reactivity was identified in 5 out of 40 serum samples obtained from febrile patients who did not have brucellosis when the fusion protein was evaluated. These samples comprised 3 instances of *Escherichia coli* infection, 1 instance of *Pseudomonas putida* infection, and 1 instance of *Streptococcus dysgalactiae* infection. In contrast, cross-reactivity with LPS was detected in 14 cases, which included 8 instances of *Escherichia coli* infection, 3 instances of *Staphylococcus aureus* infection, and 1 instance each of *Enterococcus faecium*, *Klebsiella pneumoniae*, *Moraxella osloensis*, *Pseudomonas putida*, and *Streptococcus dysgalactiae* infection. The detailed results are presented in the **Supplementary Material**.

## 4 Discussion

Indirect ELISA is endorsed by the World Organization for Animal Health (OIE) for a variety of applications, including the assessment of population freedom from infection, the determination of individual animal freedom from infection, contributions to eradication strategies, confirmation of criminal suspects or clinical cases, and the surveillance of herd or flock prevalence of infection in animals (18). However, traditional iELISAs utilized for the diagnosis of brucellosis typically employ LPS as the antigen. LPS is commonly found in gram-negative bacteria and has been demonstrated to cross-react with *Escherichia coli* O157:H7 and *Yersinia enterocolitica* O:9 when used as a diagnostic antigen (8, 9). Consequently, the identification of more specific diagnostic antigens may enhance the efficacy of iELISA applications.

Proteomics encompasses the comparative analysis of the complete set of proteins expressed by a microorganism, thereby facilitating the classification and identification of pertinent proteins that can be characterized as protein molecules (19, 20). These molecules may serve as potential targets for novel drug development or as molecular markers for the early diagnosis of diseases. In the present study, we selected both wild and vaccine strains of *Brucella* for proteomic analysis. Drawing upon the results obtained and a comprehensive review of the literature, several proteins exhibiting significant differences were identified as candidate targets.

ABC transporters are integral to the biosynthetic pathways of extracellular polysaccharides. The deletion of the ABC transporter ATPase gene has been shown to diminish the virulence of wild-type strains and enhance the resistance of mice to challenges posed by these strains, thereby underscoring its significant immunological role in wild-type strains (21). Research has established that this transporter can be utilized for immunoenzymatic assays, specifically iELISA, in the diagnosis of bovine brucellosis (22). Heat shock proteins are recognized as major antigens that play a critical role during *Brucella abortus* infection; notably, DnaK, GroEL, and GroES are three heat shock proteins identified in *Brucella*. Immunization of animals with these recombinant proteins has the potential to elicit a Th1 immune response and generate protective antibodies, thereby highlighting their applicability in the serological diagnosis of brucellosis (23–28). Malate dehydrogenase (MDH), a pivotal enzyme in the tricarboxylic acid cycle, is capable of inducing Th2-related immune responses, and recombinant MDH (rMDH) has been employed in the diagnosis of bovine brucellosis (29–31). Additionally, *Brucella* Cu-Zn superoxide dismutase (Cu-Zn SOD), a periplasmic protein, has been shown to confer protection against *Brucella abortus* infection when mice are immunized with recombinant Cu-Zn SOD protein (32, 33). Furthermore, the transcriptional regulator VjbR is essential for the interaction with host cells during *Brucella* infection and is critical for the virulence of the intracellular facultative pathogen *Brucella* (34). Animal studies have confirmed its significant immunoprotective effect against brucellosis (35, 36).

Bioinformatics technologies have been extensively utilized in the identification of disease diagnostic antigens and the development of vaccines. A variety of techniques have been employed to generate predictions for antigen epitopes. Among the most commonly used methods for predicting linear epitopes are Bepipred, ABCpred, COBEpro, and SVMTriP (14–17). Bepipred integrates hidden Markov models with propensity scale methods to predict linear B-cell epitopes. ABCpred, which is based on a neural network framework, achieves predictions with an accuracy of approximately 65.93%. COBEpro employs a two-step approach to predict linear B-cell epitopes, initially predicting short peptides using a mechanical model, followed by scoring each amino acid residue. SVMTriP, a leading method in this domain, utilizes a support vector machine model that combines tri-peptide similarity with propensity scores. When applied to non-redundant B-cell linear epitopes sourced from the Immune Epitope Database (IEDB), SVMTriP demonstrated a sensitivity of 80.1% and a precision of 55.2% through fivefold cross-validation, resulting in an area under the curve (AUC) value of 0.702. However, the accuracy of individual prediction tools is frequently suboptimal. Consequently, this study selected four prediction tools to enhance overall accuracy. We integrated immunological information parameter predictions with B-cell epitope predictions and compared the B-cell epitopes identified by the four methods. Overlapping B-cell epitopes were subsequently selected as candidate B-cell epitopes for the construction of multiepitope fusion proteins.

In this study, a total of 32 epitopes were predicted, and the constructed fusion protein, utilized as a diagnostic antigen, was compared to LPS. Although the fusion protein demonstrated slightly inferior performance, it exhibited reduced cross-reactivity, thereby highlighting its advantages in the diagnosis of brucellosis. The approach of developing multiepitope fusion proteins not only preserves the immunogenicity of individual antigenic epitopes but also significantly diminishes the cross-reactivity of the diagnostic antigen, thereby enhancing specificity and improving diagnostic accuracy. It is important to note that the sera selected for testing in the evaluation of the constructed iELISA, particularly the 96 negative samples, did not allow for the determination of whether these samples were from individuals infected with other pathogens or from healthy individuals. The evaluation indices, including sensitivity, specificity, and false-positive rates, are subject to change with variations in sample selection. In contrast, when assessing cross-reactivity, the selected sera were sourced from patients with confirmed infections from other pathogens, all of whom presented with fever symptoms necessitating differential diagnosis from brucellosis. Consequently, these sera are particularly relevant for evaluating the validity of the iELISA. LPS is known to be present in gram-negative bacteria, and its cross-reactivity with *Escherichia coli* varies. In this study, the purity of the fusion protein expressed in prokaryotic systems was only 90.1%, with some impurities originating from *E. coli*, which may contribute to its cross-reactivity with *E. coli* infections. Future efforts to enhance the specificity of the fusion protein may involve improvements in its purification process. Furthermore, the advancement of this technology presents new perspectives and methodologies for the diagnosis of brucellosis, potentially addressing the limitations of current serological diagnostic techniques. However, the assessment of the advantages and disadvantages of the multiepitope fusion protein compared to LPS based solely on the testing of this limited sample size is not reliable. While LPS demonstrates an advantage concerning false positivity, fusion proteins exhibit a benefit in terms of reduced cross-reactivity; this discrepancy may be influenced by the selection of serum samples.

Currently, there is no vaccine available for human brucellosis, which renders the findings of this study inconclusive in determining whether this protein can effectively differentiate between sera from naturally infected individuals and those who have been immunized with a vaccine. Additional research is necessary to obtain sera from both immunized and naturally infected animals for further testing.

## 5 Conclusion

In summary, a novel multiepitope fusion protein, developed utilizing bioinformatics and TMT proteomics technology, has demonstrated significant potential as a diagnostic antigen for brucellosis, exhibiting high sensitivity and specificity. This innovative approach marks a considerable advancement in the field of brucellosis diagnosis, providing a more accurate and reliable alternative to existing methodologies. However, this study is not without its limitations. The sample size, comprising 100 positive and 96 negative serum samples, is relatively small, which may hinder the ability to make robust claims regarding diagnostic efficacy, particularly concerning the generalizability of results to diverse populations or various geographic regions where brucellosis is endemic. Additionally, it is crucial to consider the subcellular localization of candidate proteins, as this factor may significantly influence the immunogenicity of the fusion protein. Furthermore, the validity of the constructed iELISA method necessitates future comparisons with commercially available kits, and further validation through larger-scale clinical trials is essential to confirm the diagnostic performance of this novel antigen.

## Data Availability

The original contributions presented in the study are included in the article/**Supplementary Material**. Further inquiries can be directed to the corresponding authors.

## Supplementary material

Supplementary Material 1 Sheet 1, OD_450_ of positive sera; sheet 2, OD_450_ of negative sera; sheet 3, data for Cross-Reactivity Assessment, sheet 3, 152 highly expressed proteins in *Brucella* wild strain.

Supplementary Material 2 Results of TMT-Proteomics quality control analysis.
