# Supplementary material for: Preparation and application of a Brucella multiepitope fusion protein based on bioinformatics and Tandem Mass Tag-based proteomics technology
Source: Front Immunol. 2025 Jan 10;15:1509534. doi: 10.3389/fimmu.2024.1509534 (PMC11757136; doi:10.3389/fimmu.2024.1509534)
Supplement: Supplementary Material 1 — Sheet 1, OD450 of positive sera; sheet 2, OD450 of negative sera; sheet 3, data for Cross-Reactivity Assessment, sheet 3, 152 highly expressed proteins in Brucella wild strain. [file DataSheet1.docx]

**Preparation and application of a *Brucella* multiepitope fusion protein based on bioinformatics and Tandem Mass Tag-based proteomics technology**

1-1 1-2 1-3 1-4 1-5 1-6 M


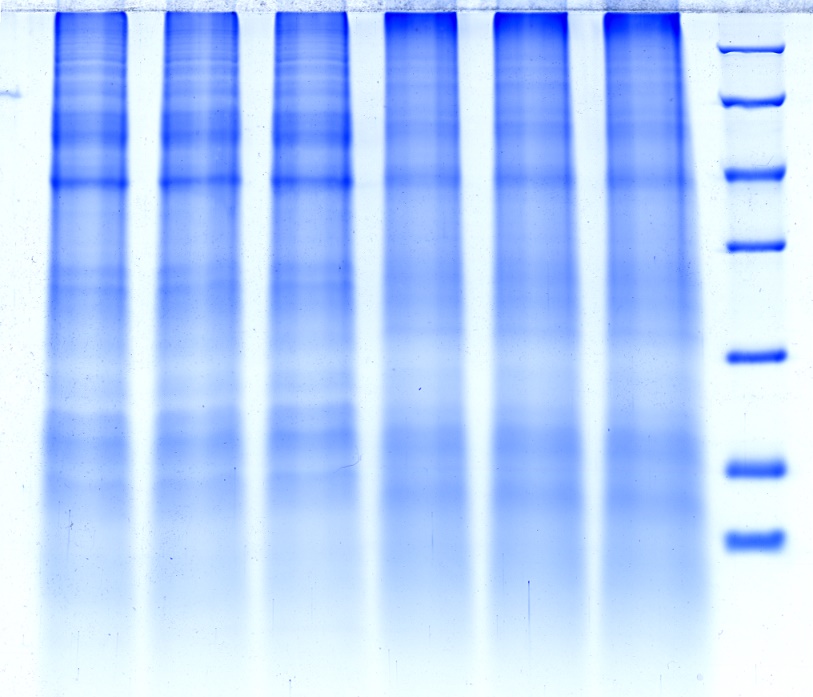


14 kD

18 kD

25 kD

35 kD

45 kD

66 kD

116 kD

**Figure S1. 12% SDS-PAGE of protein expression for TMT-Proteomics quality control analysis.** M: Marker, Lanes 1-1 to 1-3, 3 parallel samples of vaccine *Brucella* strain, Lanes 2-1 to 3-3, 3 parallel samples of wild-type *Brucella* strains

**Table S1. Quantitative results of protein samples from bacterial samples(Bradford)**

| Sample | 1-1 | 1-2 | 1-3 | 2-1 | 2-2 | 2-3 |
| --- | --- | --- | --- | --- | --- | --- |
| Concentration  (μg/μL) | 1.11 | 1.27 | 1.15 | 1.76 | 1.91 | 1.87 |
| Sample volume  (μL) | 200 | 200 | 200 | 200 | 200 | 200 |
| Total amount of protein（μg） | 223 | 255 | 231 | 353 | 383 | 374 |
